# Effect of Repeated Intravitreal Ranibizumab and Aflibercept Injections on the Cornea in Patients with Age-Related Macular Degeneration

**DOI:** 10.1155/2020/4928905

**Published:** 2020-06-09

**Authors:** Beata Urban, Magdalena Szwabowicz, Alina Bakunowicz-Łazarczyk

**Affiliations:** ^1^Department of Pediatric Ophthalmology and Strabismus, Medical University of Bialystok, Waszyngtona 17, 15-274 Bialystok, Poland; ^2^Department of Ophthalmology, Municipal Hospital in Olsztyn, Niepodległości 44, 10-045 Olsztyn, Poland

## Abstract

**Purpose:**

To assess the effect of repeated intravitreal ranibizumab injections (RI) and aflibercept injections (AI) on the corneal endothelium and central corneal thickness (CCT) in patients with age-related macular degeneration (AMD).

**Materials and Methods:**

In the retrospective study, 110 eyes of 106 patients, aged 52 to 93 years, were analyzed. Fifty eyes were treated only with RI (I group), and 60 eyes were treated only with AI (II group). Every patient received one intravitreal injection of 0.5 mg of ranibizumab once a month or 2 mg of aflibercept for 3 subsequent months. Each patient received only 3 injections during the whole observation period. Corneal analysis was obtained with the specular microscope. Examinations were performed before initial treatment, after each injection, and 6 months after the first injection. Analysis included corneal endothelial cell density (ECD), hexagonal cell percentage (% Hex), coefficient of variation (CoV), and CCT.

**Results:**

There was a statistically significant ECD loss, regardless of the type of the anti-VEGF agent. The mean ECD value in the I group was 2397 ± 459 cells/mm^2^ before RI, 2389 ± 459 cells/mm^2^ after the first RI, 2386 ± 467 cells/mm^2^ after the second RI, 2378 ± 475 cells/mm^2^ after the third RI, and 2357 ± 460 cells/mm^2^ 6 months after the first RI. The mean ECD value in the II group was 2448 ± 493 cells/mm^2^ before treatment, 2456 ± 498 cells/mm^2^ after the first AI, 2426 ± 496 cells/mm^2^ after the second AI, 2402 ± 488 cells/mm^2^ after the third AI, and 2348 ± 473 cells/mm^2^ 6 months after the first AI. In comparison with the group treated with RI, the group treated with AI presented a greater ECD loss at each measuring point. The percentage of hexagonal cells was decreased in both groups. There was a slight increase in polymegathism in both treated groups. Ranibizumab proved to cause a small increase in CCT, while CCT remained unchanged in the aflibercept group.

**Conclusions:**

Repeated intravitreal injections of 0.5 mg of ranibizumab or 2 mg of aflibercept can influence the morphology of the corneal endothelium but not CCT.

## 1. Introduction

Age-related macular degeneration (AMD) is one of the most common causes of permanent visual impairment and blindness in developed countries among people over 60 years of age. It is estimated that the number of people with AMD worldwide will be 288 million in 2040 [1].

Intravitreal administration of vascular endothelial growth factor antagonists (anti-VEGF), mainly ranibizumab and aflibercept, became the gold standard of modern wet AMD therapy [2, 3]. Ranibizumab (Lucentis, Genentech, South San Francisco, CA), a humanized monoclonal antibody fragment, was the first anti-VEGF agent shown to improve visual acuity in patients with wet AMD, and it was approved for use in wet AMD in Europe in 2007 [4]. Aflibercept (Eylea; Regeneron, Tarrytown, New York, USA) is an antivascular endothelial growth factor agent that binds to all vascular endothelial growth factor-A and vascular endothelial growth factor-B isoforms and also placental growth factors 1 and 2 [5, 6]. Intravitreal aflibercept (IVT-AFL) is used in wet AMD in Europe since 2012. Several clinical studies confirmed high safety profile of anti-VEGF agents, but still, there are only few reports concerning potentially harmful effects of these substances on the corneal endothelium. The necessity of recurring intravitreal injections may additionally increase the likelihood of side effects. The question, whether repeated intravitreal injections of ranibizumab or aflibercept may adversely affect the corneal endothelium and corneal thickness, is still valid.

The aim of this study was morphometric analysis and comparison of the corneal endothelium and central corneal thickness in patients with age-related macular degeneration treated with repeated intravitreal ranibizumab or aflibercept, using specular microscopy. Analysis included corneal endothelial cell density (ECD), hexagonal cell percentage (% Hex), coefficient of variation (CoV), and central corneal thickness (CCT). It was crucial to compare these parameters between the group of patients treated with ranibizumab and the group of patients treated with aflibercept.

## 2. Materials and Methods

In this retrospective study, 110 eyes of 106 patients (both males and females) were analyzed. All patients were treated in the Department of Ophthalmology, Municipal Hospital in Olsztyn, Poland. The study was approved by the bioethical committee in Bialystok Medical University and was conducted in accordance with the tenets of the Declaration of Helsinki. All patients signed a consent form before their inclusion in the study. Each patient was qualified for anti-VEGF treatment due to active wet AMD. For the subsequent three months, every patient received one intravitreal injection of 0.5 mg of ranibizumab or 2 mg of aflibercept once per month. Each patient received only three injections during the whole observation period.

**Figure 1:**
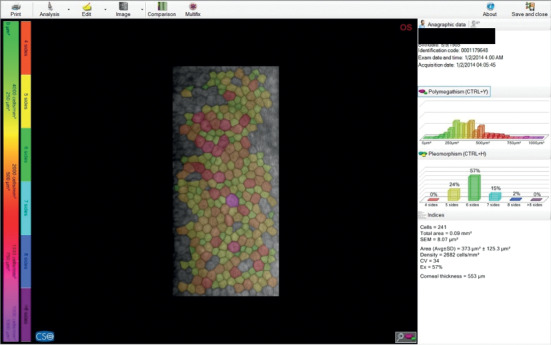
Specular photomicrograph from the CSO SP-02 noncontact specular microscope.

Specular microscopy of the central cornea was performed by a noncontact specular microscope CSO SP-02 (CSO, Italy) (Figure 1). Examinations were performed before initial treatment, after each of three subsequent ranibizumab/aflibercept injections, and finally, six months after the first intravitreal injection.

The inclusion criterion was active wet AMD, never treated before except for oral vitamins and mineral supplementation. The exclusion criteria comprised past intraocular surgery in the treated eye, glaucoma, pseudoexfoliation syndrome, diabetes mellitus, connective tissue disease, and use of contact lenses.

Ophthalmologic examination prior to the first intravitreal injection included best corrected visual acuity, intraocular pressure, slit-lamp examination, binocular indirect ophthalmoscopy fundus examination, and optical coherent tomography. The specular microscope was used in the automatic release mode to reduce operator-dependent variables. The operator focused and aligned a real-time image of the participant's eye. The instrument captured the endothelium in the central corneal area. Instrument software automatically calculated the quality and reliability of a captured image. If the quality of the image was poor, the measurement was repeated until the best image with good contrast was accepted. To evaluate the corneal endothelium, the endothelial cells on the 0.54 mm × 0.27 mm diameter area of the center cornea were analyzed. Regarding the number of cells counted, the different number of cells was calculated with every measurement, but the analyzed area of the center cornea was always the same. Using a built-in program in the noncontact specular microscope, the cell density (ECD), the coefficient of variation of the cell area (CoV), and the percentage of hexagonal cells (% Hex) of the corneal endothelium were evaluated. The examination was repeated from 5 to 7 days after each of the three subsequent ranibizumab/aflibercept injections and finally, six months after the first intravitreal injection.

## 3. Statistical Analysis

Statistical analysis was performed using the Statistica 12 PL programme. The Shapiro–Wilk test was used to examine the distribution of variables, and the Levene test analyzed their homogeneousness of variances. Student's *t*-test was used to compare the quantitative variables with the normal distribution. Wilcoxon test was used to compare the quantitative variables without the normal distribution.

## 4. Results

The study group included 110 patients with a mean age of 73.45 years (range, 52–93 years). All patients were phakic. Fifty eyes of 50 patients were treated only with 0.5 mg ranibizumab (first group), and 60 eyes of 56 patients were treated only with 2 mg aflibercept (second group). Mean age in the first group was 72.36 years, and mean age in the second group was 74.36 years.

Analysis of endothelial cell density (ECD) before, during, and after the treatment of AMD with ranibizumab injections is presented in Table 1. The mean ECD before RI was 2397.14 ± 459.33 cells/mm^2^, and it was significantly lower after each RI. The loss of the corneal endothelium cells was 0.3% after the first RI (Wilcoxon test, *p*=0.022), 0.5% after the second RI (Wilcoxon test, *p*=0.041), 0.8% after the third RI (Wilcoxon test, *p*=0.0007), and 1.7% 6 months after the first RI (Wilcoxon test, *p*=0.000019) (Table 2). In patients treated with aflibercept injections, ECD before the therapy was 2488.32 ± 492.88 cells/mm^2^, and similarly to RI, it was significantly reduced after each AI (Table 3). The loss of the corneal endothelium cells was 1.3% after the first AI (Wilcoxon test, *p* < 0.0001), 2.5% after the second AI (Wilcoxon test, *p* < 0.0001), 3.5% after the third AI (Wilcoxon test, *p* < 0.0001), and 5.6% 6 months after the first RI (Wilcoxon test, *p* < 0.0001) (Table 2).

Changes in the percentage of hexagonal cells (% Hex) in patients with AMD, treated with ranibizumab injections, are featured in Table 1. The decline of the percentage of hexagonal cells was noticed after each RI and 6 months after the first RI. These differences were statistically significant (Wilcoxon test: *p*=0.004; *p* < 0.0001; *p* < 0.0001; and *p* < 0.0001, respectively) (Table 2). A similar measurement, as far as the percentage of hexagonal cells is concerned, was observed after the aflibercept injections (Table 3). The decrease of % Hex after each AI was statistically significant (Wilcoxon test: *p*=0.038; *p*=0.024; *p*=0.002; and *p*=001, respectively) (Table 2).

The study of the coefficient of variation of cell size (CoV) after intravitreal injections is presented in Tables 1 and 3. A statistically significant increase of CoV was observed after the second RI, third RI, and 6 months after the first RI (Wilcoxon test: *p* < 0.0001; *p*=0.0037; and *p*=0003, respectively) (Table 2). CoV was significantly increased after the third AI and 6 months after the first AI (Wilcoxon test: *p*=0.007; *p*=0.0037; and *p*=008, respectively) (Tables 2 and 3).

The assessment of the central corneal thickness (CCT) after intravitreal injections is presented in Tables 1 and 3. The mean CCT value was 555.72 *µ*m after the second RI and 555 *µ*m 6 months after the first RI, and these differences were statistically significant (Student's *t*-test: *p*=0.028 and *p*=0.038, respectively). Changes of CCT after repeated aflibercept injections were not significant.

## 5. Discussion

Currently, the standard treatment of neovascular age-related macular degeneration (AMD) is inhibitors against vascular endothelial growth factor (VEGF), which is a major contributor to the pathogenesis of wet AMD [7]. Treatments with anti-VEGF drugs, which are delivered by frequent intravitreal injections, are now administered to millions of people annually around the world. Ranibizumab (Lucentis, Genentech, Inc., USA) was the first anti-VEGF drug approved in 2005 for wet AMD treatment [8]. It is a humanized monoclonal antibody fragment directed toward all isoforms of VEGF-A. The binding of ranibizumab to all human VEGF-A isoforms prevents the dimerization with the VEGF receptors on cell surfaces (VEGFR1 and VEGFR2), reducing angiogenesis [9]. Aflibercept (Eylea, Regeneron Pharmaceuticals, Inc., Tarrytown, NY, USA), the newest anti-VEGF agent, was approved by the FDA for the treatment of wet AMD in 2011 [10]. Aflibercept is the recombinant fusion protein, which is composed of VEGF-binding receptors 1 and 2, fused to the Fc portion of human IgG [11]. Intravitreal drugs are eliminated posteriorly across the blood-retina barrier and anteriorly via aqueous humor outflow. However, many experimental studies have shown that anti-VEGF agents are eliminated nearly completely via the anterior route [12–14]. There are no available data on the vitreous levels of ranibizumab and aflibercept after intravitreal injection in humans; therefore, vitreous half-life values are also not available. At the same time, the aqueous half-life of intravitreal ranibizumab 0.5 mg is 7.19 days in human nonvitrectomized eyes [15]. Until now, there are no available data on the half-life of aflibercept in the human eye. The most recent study of Do et al. has shown that the half-life of aflibercept in the aqueous humor after a single intravitreal injection in five patients with neovascular AMD was 11 days [16].

Intravitreal anti-VEGF therapy seems safe in the general population enrolled into numerous clinical trials, but it is important to take into account any possible local and systemic adverse events in patients receiving intravitreal anti-VEGF injections. Because anti-VEGF treatment is potentially required for years, each intravitreal injection poses a risk of uveitis, postinjection endophthalmitis, retinal tear or detachment, vitreous hemorrhage, sustained elevated intraocular pressure, cataract progression, pain, and vitreous floaters [17, 18]. The risk of ocular complications is significantly higher for patients undergoing anti-VEGF injection when compared to people with wet AMD who did not receive anti-VEGF treatment [17].

Treatment of wet AMD requires repeated intravitreal injections, and intravitreal agents are eliminated from the eye almost exclusively via the aqueous humor outflow, so the accompanying risk of corneal endothelium impairment should be considered. There are few reports involving the influence of intracameral aflibercept and ranibizumab on the corneal endothelium [19–21]. Ari et al. examined the effect of intracameral ranibizumab on the rabbit corneal endothelium by scanning electron microscopy [21]. They observed the deterioration in endothelial cell morphology after intracameral injection of 1 and 0.5 mg ranibizumab. Gharbiya et al. demonstrated that anterior chamber injection of two different anti-VEGF agents, aflibercept and ranibizumab, affects the rabbit corneal endothelium in terms of survival and apoptosis and is associated with changes in the endothelial expression of the NGF precursor (proNGF) and p75 neurotrophin receptor (p75NTR) [19]. On the contrary, Wilhelm et al. observed that ranibizumab and bevacizumab have no damaging effects on the corneal endothelium after injection into the anterior chamber in a porcine eye model [20]. It is in agreement with the study of Merz et al. who did not show cytotoxic effects of intracameral application of ranibizumab on the human endothelium in the corneal donor tissue, but in the authors' opinion, no conclusions on potential long-term effects can be drawn [22]. Still, there are limited data available on the effects of repeated anti-VEGF injection on the human corneal endothelium, considering that antiangiogenic substances are in direct contact with the sensitive corneal endothelium.

In this retrospective study, we analyzed corneal endothelium cells and corneal thickness in two groups of patients: first group (50 eyes of 50 patients) treated only with ranibizumab and second group (60 eyes of 56 patients) treated only with aflibercept. Each patient received 3 monthly doses of intravitreal injection of 0.5 mg ranibizumab or 2 mg of aflibercept.

Endothelial cell density (ECD), percentage of hexagonal cells (pleomorphism), coefficient of variation of the cell size (polymegathism), and central corneal thickness (CCT) were evaluated before treatment, after every 3 monthly injections, and 6 months after the first injection.

Mean value of ECD in patients treated with ranibizumab injections (RI) was 2397 ± 459 cells/mm^2^ before treatment, 2389 ± 459 cells/mm^2^ after the first RI, 2386 ± 467 cells/mm^2^ after the second RI, 2378 ± 475 cells/mm^2^ after the third RI, and 2357 ± 460 cells/mm^2^ 6 months after the first RI. The decrease of ECD was 0.3% after the first RI, 0.5% after the second RI, 0.8% after the third RI, and 1.7% 6 months after the first RI, and these differences were statistically significant (Wilcoxon test, *p*=0.022; *p*=0.041; *p*=0.0007; and *p*=0.000019, respectively). In the study by Benítez-Herreros et al., they observed similar endothelium cell loss (1.8%) 6 months after the first RI in patients with AMD [23]. They concluded that repeated intravitreal injections of 0.5 mg ranibizumab do not seem to cause substantial changes in the corneal endothelium. Similar results were obtained by Joshi et al. who noted that there was no significant change in endothelial cell density after intravitreal ranibizumab [24]. In their study, the mean value of ECD in phakic eyes on days 1, 7, and 30 after RI was 2314.51 ± 212.08, 2313.92 ± 212.7, and 2313.63 ± 216.86 cells/mm^2^, respectively. Arslan et al. measured ECD in patients who received only a single intravitreal anti-VEGF injection for two consecutive months [25]. They found that mean ECD for phakic eyes was significantly lower one month after the first (*p*=0.047) and second injections (*p*=0.034) compared to the values at preinjection levels. Different observations were done by Ho et al. who have shown that EDC was 2233.22 cells/mm^2^ prior to the ranibizumab injections and 2465.89 cells/mm^2^ 3 months after the injections [26]. Benítez-Herreros et al. analyzed the effect of intravitreal ranibizumab on the corneal endothelium in the treatment of AMD, and they observed no significant difference in the endothelial cell count before injection or 6 months after the first RI [23].

In the current study, the mean value of ECD in patients treated with aflibercept injections (AI) was 2448 ± 493 cells/mm^2^ before treatment, 2456 ± 498 cells/mm^2^ after the first AI, 2426 ± 496 cells/mm^2^ after the second AI, 2403 ± 488 cells/mm^2^ after the third AI, and 2348 ± 473 cells/mm^2^ 6 months after the first AI. The decline of ECD was 1.3% after the first AI, 2.5% after the second AI, 3.5% after the third AI, and 5.6% 6 months after the first AI, and it was statistically significant (Wilcoxon test, *p* < 0.0001; *p* < 0.0001; *p* < 0.0001; and *p* < 0.0001, respectively). All values of ECD in patients treated with AI are within normal limits, but it is worth emphasizing that the loss of endothelial cells in patients treated with AI was greater during each control in comparison with the loss of endothelial cells in patients treated with RI. We are aware that endothelial cell loss in the aflibercept group after 6 months is quite sizable (5.6%). In our opinion, advanced age has an important impact on ECD loss in the AI group. Among 60 patients, 38 (63.3%) of them were over 70 years and 20 (33.3%) were over 80 years. When it comes to the ranibizumab group, among 50 patients, 28 (56%) were over 70 years and only 9 (18%) were over 80 years. Another possible reason of decreasing rate of ECD in the AI group can be high incidence of IOP elevation. In the current study, intraocular pressure was not measured immediately after injection but after 5–7 days. A theoretical risk of incidental ocular hypertension in the first day after aflibercept injection cannot be ruled out. Contrary to our results, the review study has shown that repeated intravitreal aflibercept injections for 52 weeks had no apparent corneal endothelial toxicity noted on specular microscopy in patients treated for AMD [27]. In this study, endothelial cell density was 2410 ± 364 versus 2388 ± 384 cells/mm at baseline and remained unchanged at 2401 ± 353 versus 2376 ± 364 cells/mm at 52 weeks (*p*=0.87). On the contrary, the values of ECD in the review study and in the current study are still within normal limits. Certainly, further studies are required.

In our patients, the mean percentage of hexagonal cells (pleomorphism) in the group treated with ranibizumab was 53.7 ± 9% before treatment, 51.6 ± 8% after the first RI, 50.6 ± 8% after the second RI, 49.8 ± 9% after the third RI, and 49.2 ± 9% 6 months after the first RI. These differences were statistically significant (Wilcoxon test, *p*=0.004; *p* < 0.0001; *p* < 0.0001; and *p* < 0.0001, respectively). Our results are in contrast to other studies, in which there were no significant differences in this parameter [23]. We have observed similar statistically significant differences in the group treated with aflibercept. The mean percentage of hexagonal cells was 52.3 ± 9%, 51.5 ± 8%, 51.4 ± 7%, 50.4 ± 8%, and 49.3 ± 8% (Wilcoxon test, *p*=0.038; *p*=0.024; *p*=0.002; and *p*=0.001, respectively). In the studies of Lass et al. and Muto et al., the percentage of hexagonal cells after repeated aflibercept injection remained unchanged [28, 29]. In the current study, increase of pleomorphism was noticed regardless of the type of the anti-VEGF agent. Different observations were done by other authors, who have shown no changes of pleomorphism after both RI and AI [23, 27, 28].

The coefficient of variation of the cell size (polymegathism) in the group treated with ranibizumab was 36.7 ± 6% before treatment, 37.1% ± 6 after the first RI, 38.8 ± 6% after the second RI, 37.7 ± 6% after the third RI, and 38.1 ± 6% 6 months after the first RI. Significant increase of polymegathism was noticed after the second and third RI and 6 months after the first RI (Wilcoxon test, *p* < 0.0001; *p*=0.0037; and *p*=0.0003, respectively). In the second group of patients, polymegathism was significantly higher after the third AI and 6 months after the first AI: 37.1 ± 5% and 37.6 ± 6% (Wilcoxon test, *p*=0.007; *p*=0.008, respectively). The coefficient of variation of the cell size in most studies concerning anti-VEGF therapy remained unchanged [23, 27, 28].

In the present study, we have also analyzed central corneal thickness (CCT). In patients treated with RI, CCT before treatment was 551.1 ± 34 *µ*m. Significant increase of CCT was observed after the second RI (555.7 ± 35 *µ*m) and 6 months after the first RI (555 ± 36 *µ*m) (Student's *t*-test, *p*=0.028 and *p*=0.038, respectively). The final CCT in this group of patients was only 4 *µ*m thicker when compared with the values of CCT before treatment. As regards CCT of the patients treated with AI, there were no significant differences in CCT after aflibercept therapy. Similar results were obtained by other authors [23, 24, 28].

Potential limitations of our study should be mentioned: firstly, a short follow-up period (six months), secondly, there were only three intravitreal injections, and finally, the small sample size. Certainly, longitudinal studies involving large sample numbers of patients treated with both anti-VEGF agents are required. Finally, in the current study, we present data on automated endothelial cell counts. Automated endothelial cell analysis is usually characterized by high variability and reduced reproducibility.

## 6. Conclusions

In conclusion, in the current study, statistically significant corneal endothelial cell loss was shown, regardless of the type of the intravitreal anti-VEGF agent. In comparison with the group treated with ranibizumab, the group treated with aflibercept presented greater endothelial cell density loss at each measuring point. The percentage of hexagonal cells was decreased in both groups. This study also showed slight increase in polymegathism in both treated groups. Ranibizumab proved to cause small but not significant increase in central corneal thickness, while central corneal thickness remained unchanged in the aflibercept group.

## Figures and Tables

**Table 1:** Morphometric analysis of the corneal endothelial cell density (ECD), the percentage of hexagonal cells (% Hex), the coefficient of variation of cell size (CoV), and central corneal thickness (CCT) in the treated eye before and after ranibizumab injections (RI).

Ranibizumab injection (RI)					
	Before 1^st^ RI Mean ± SD	After 1^st^ RI Mean ± SD	After 2^nd^ RI Mean ± SD	After 3^rd^ RI Mean ± SD	6 m after 1^st^ RI Mean ± SD
ECD (cells/mm^2)^	2397.1 ± 459.3	2389.4 ± 459.2	2386.1 ± 467.5	2378.1 ± 475.3	2356.8 ± 460.4
% Hex	53.7 ± 8.6%	51.6 ± 8.5%	50.6 ± 8.1%	49.8 ± 9.1%	46.2 ± 8.9%
CoV	36.7 ± 6.3	37.1 ± 5.7	38.8 ± 6.6	37.7 ± 6.4	38.1 ± 6.3
CCT (*µ*m)	551.1 ± 33.7	551.1 ± 31.2	555.7 ± 34.8	552.7 ± 32.7	555.1 ± 36.2

**Table 2:** Wilcoxon test for the endothelium cell density (ECD), the percentage of hexagonal cells (% Hex), and the coefficient of variation of cell size (CoV) before and after ranibizumab injections (RI) and aflibercept injections (AI).

	Ranibizumab injections (RI)	Aflibercept injections (AI)
	*p* value	*p* value
ECD before and after 1^st^ injection	0.022	<0.0001
ECD before and after 2^nd^ injection	0.041	<0.0001
ECD before and after 3^rd^ injection	0.0007	<0.0001
ECD before and 6 months after 1^st^ injection	0.000019	<0.0001
% Hex before and after 1^st^ injection	0.0043	0.038
% Hex before and after 2^nd^ injection	0.000002	0.024
% Hex before and after 3^rd^ injection	<0.0001	0.0018
% Hex before and 6 months after 1^st^ injection	<0.0001	0.00091
CoV before and after 1^st^ injection	0.14	0.72
CoV before and after 2^nd^ injection	0.000004	0.60
CoV before and after 3^rd^ injection	0.002	0.0073
CoV before and 6 months after 1^st^ injection	0.0003	0.0083

**Table 3:** Morphometric analysis of the corneal endothelial cell density (ECD), the percentage of hexagonal cells (% Hex), the coefficient of variation of cell size (CoV), and central corneal thickness (CCT) in the treated eye before and after aflibercept injections (AI).

Aflibercept injection (AI)					
	Before 1^st^ AI Mean ± SD	After 1^st^ AI Mean ± SD	After 2^nd^ AI Mean ± SD	After 3^rd^ AI Mean ± SD	6 m after 1^st^ AI Mean ± SD
ECD (cells/mm^2)^	2488.3 ± 492.9	2455.7 ± 497.7	2425.5 ± 496.1	2402.3 ± 488.5	2348.1 ± 473.5
% Hex	52.6 ± 8.7	51.5 ± 7.9	51.4 ± 7.1	50.4 ± 7.9	49.3 ± 7.8
CoV	36.1 ± 6.7	366.3 ± 5.9	36.4 ± 5.8	37.1 ± 5.3	37.6 ± 5.5
CCT (*µ*m)	556.1 ± 40.7	559.9 ± 41.4	555.6 ± 43.1	555.3 ± 45.9	552.1 ± 48.2

## Data Availability

The data used to support the findings of this study are available from the second author upon request.
